# Regenerative Role of T Cells in Nerve Repair and Functional Recovery

**DOI:** 10.3389/fimmu.2022.923152

**Published:** 2022-07-05

**Authors:** Xiaoxuan Tang, Qiaoyuan Li, Tingting Huang, Han Zhang, Xiaoli Chen, Jue Ling, Yumin Yang

**Affiliations:** ^1^ Key Laboratory of Neuroregeneration, Ministry of Education and Jiangsu Province, Co-Innovation Center of Neuroregeneration, Jiangsu Clinical Medicine Center of Tissue Engineering and Nerve Injury Repair, Nantong University, Nantong, China; ^2^ Medical School of Nantong University, Nantong University, Nantong, China

**Keywords:** T cells, immune response, nerve regeneration, neuroinflammation, nerve injury

## Abstract

The immune system is essential in the process of nerve repair after injury. Successful modulation of the immune response is regarded as an effective approach to improving treatment outcomes. T cells play an important role in the immune response of the nervous system, and their beneficial roles in promoting regeneration have been increasingly recognized. However, the diversity of T-cell subsets also delivers both neuroprotective and neurodegenerative functions. Therefore, this review mainly discusses the beneficial impact of T-cell subsets in the repair of both peripheral nervous system and central nervous system injuries and introduces studies on various therapies based on T-cell regulation. Further discoveries in T-cell mechanisms and multifunctional biomaterials will provide novel strategies for nerve regeneration.

## Introduction

Human nerves are divided into two main types: the peripheral nervous system (PNS) and the central nervous system (CNS), including the brain and spinal cord (1–3). PNS and CNS injuries are often caused by traffic accidents, natural disasters, war damage, and iatrogenic side effects of surgery (4). Due to the complexity of the pathological process and poor regrowth capacity, nerve injury becomes one of the most serious traumas and usually causes long-term disability and death in humans. Severe damage to the central nervous system can even lead to paralysis, seriously affecting the physical and mental health of individuals and causing significant social and economic problems (5). Unfortunately, despite much effort being made to address this urgent clinical need, effective treatments for traumatic brain injury (TBI) and spinal cord injury (SCI) are still elusive (6–8).

In the early clinical treatment of nerve injury, allografting was considered one of the most effective ways to bridge the injury gap, as it elicits a foreign body immune response to avoid graft failure (9, 10). Therefore, it is generally believed that inflammation is detrimental to the repair of nerve damage, and efforts have been made to reduce or eliminate the immune response by using immunosuppressive agents (11–13). However, as research has progressed, it found that the immune response to CNS/PNS injury can be both neuroprotective and neurodestructive (14–16).

Therefore, in the clinical management of treating neurotrauma, it is of paramount importance to be able to expand the immune response that promotes tissue repair while inhibiting excessive inflammation that leads to further damage (17). At present, research on the immune system and the repair of nerve injury mainly focuses on macrophages (18, 19). For instance, in the early stage of nerve injury, phagocytes represented by macrophages are rapidly recruited to the injury site and activated. During inflammation, immunostimulatory phenotype (M1) macrophages remove myelin debris and pathogens through phagocytosis and autophagy to promote nerve growth (20). Then, anti-inflammatory immunosuppressive (M2) macrophages can secrete immunoregulatory cytokines such as interleukin 14 (IL-4), interleukin 10 (IL-10), and interleukin 13 (IL-13), which promote angiogenesis and matrix formation (21). However, although T cells are regarded as important members involved in the immune response of the tissue repair process, relatively few studies have focused on how T cells affect nerve regeneration. Thus, this review mainly discusses the role of T cells in the repair of nerve injury and introduces studies on the treatment of nerve injury by regulating T cells.

## T Cells in Nerve Injury

T cells are a heterogeneous cell population, and there are various classification methods. According to the difference in cell surface differentiation antigen (CD), they can be divided into two main subgroups, CD4^+^ and CD8^+^ (22). Additionally, gamma-delta (γδ) T cells are another subset of T cells that can facilitate the inflammatory responses of lymphoid and myeloid lineages, especially in the initial immune responses (23). The inflammatory response is essential in the whole pathological process of nerve regeneration, and CD4^+^ T cells, such as T helper cells and regulatory T cells, are significant in the adaptive immunity of nerve repair (24, 25).

### Th Cells in the Pathological Process After Injury

T helper (Th) cells have been widely recognized as one of the essential members of both innate and adaptive immune responses, which also contribute to neutrophilic inflammation, remyelination, and neuropathic pain after nerve injury (26–28). Th cells can be divided into several subsets, such as type I Th (Th1) cells, type II Th (Th2) cells, and type 17 Th (Th17) cells (26). Generally, Th1 cells secrete interferon-gamma (IFN-γ) to activate M1 macrophages in acute inflammatory responses, whereas Th2 cells produce anti-inflammatory cytokines such as IL-4 and IL-13 to promote M2 polarization of macrophages to improve repair (29). Lymphocyte polarization from the Th1 to Th2 phenotype during nerve regeneration improves functional recovery and myelination (30). In mechanical lesions of the CNS or stroke, a systemic Th2 shift is important in immunomodulation to facilitate regeneration and prevent autoimmune disease by downregulating Th1- or Th17-driven cellular immune responses (31).

#### Remyelination

In PNS injuries, Wallerian degeneration occurs at a very early stage after injury, which then triggers a cascade of inflammatory responses to clear debris and alter the microenvironment to support axon regrowth in the peripheral nerve (32). Although T cells only infiltrate the injured sites by 3 days after injury, these cells can produce various cytokines to shift the later phase of the immune response, and both Th1 and Th2 cells have been reported to promote typical nerve regeneration (33). For CNS injuries, increasing evidence has shown that Th cells initially infiltrate into the cerebrospinal meninges during immune responses in the CNS (34). Remyelination of newborn or sparing axons is essential for nerve function recovery (35). The myelination of Schwann cells or oligodendrocytes on regenerated axons can facilitate electrical impulses and secrete neurotrophic factors to promote axon repair (28, 36). In fact, evidence has indicated that Th cells may significantly modulate the remyelination process. For instance, *in vitro* studies have shown that the supernatants of Th1 or Th17 cells are cytotoxic to human fetal oligodendrocyte progenitor cells (OPCs) and reduce the differentiation of OPCs into ${\text{O}}_{4}^{+}$ cells *via* proinflammatory cytokines such as tumor necrosis factor-α (TNF-α) or astrocyte-derived CXCL10 (37). Furthermore, Baxi’s study demonstrated that both Th1 and Th17 cells infiltrated into the CNS of mice with demyelinating disease, which inhibited endogenous remyelination in the CNS by secreting IFN-γ and interleukin 17 (IL-17) (38). These studies have shown that Th1 and Th17 appear to inhibit the remyelination process to hinder functional recovery.

#### Neuropathic Pain

Many clinical data have shown that Th cells are also important in neuropathic pain after injury (39). Recent studies have indicated increased numbers of Th1 or Th17 cells in patients with neuropathic pain, which are also positively correlated with pain intensity (40, 41). Further studies revealed that the ratio of inflammatory Th17 cells to Treg cells was significantly reduced in patients with chronic neuropathic pain (42). To date, there is still no consensus on the pathophysiological mechanisms of pain. Further studies on the mechanism of T cells and neuropathic pain have shown that the peripheral enzymatic activity of cathepsin S is essential for the antigen-specific activation of CD4^+^ T cells after peripheral nerve injury, and then the activated T cells infiltrate into the spinal cord and secrete IFN-γ to reactivate microglia to contribute to the development of neuropathic pain (43). Hartlehnert’s study demonstrated that Schwann cells expressed MHC-II molecules to activate Th cells, which further promoted posttraumatic axonal loss and neuropathic pain after peripheral nerve injury (44). In addition, Treg cells can also infiltrate and proliferate into the injury site and suppress the Th1 response *via* IL-10 signaling to inhibit the development of neuropathic pain (45).

### Treg Cells in the Pathological Process After Injury

Regulatory T cells (Treg cells) are also a subset of CD4^+^ T lymphocytes, which are characterized by the cell surface markers CD4 and CD25 and the transcription factor forkhead box protein P3 (FOXP3) (46, 47). By suppressing the activation of other immune cells, Treg cells can maintain immune homeostasis and mediate immune tolerance in the inflammatory response.

#### Immunosuppression

Since excessive neuroinflammation can hinder axonal regeneration and functional recovery after nerve injuries, the immunosuppressive properties of Treg cells have been considered to improve the healing process (48). Zhou’s study indicated that Treg cells have a strong capacity to modulate immune responses in the brain by promoting the anti-inflammatory phenotype polarization of microglia and macrophages *via* IL-10 after intracerebral hemorrhage-induced injury (49). Li’s clinical study found that the levels of circulating Tregs in survival were significantly higher than those in nonsurvival TBI patients (50). Cao’s study found increased levels of Treg cells in the CNS of mice with TBI treated with fingolimod, which contributed to functional recovery, brain edema, and blood–brain barrier (BBB) healing (51). Moreover, Treg cells can not only suppress the secretion of matrix metalloproteinase 9 from neutrophils but also inhibit the production of CC-chemokine ligand 2 from endothelial cells to protect the blood–brain barrier after stroke (52).

#### Remyelination and Neuroprotection

**Figure 1:**
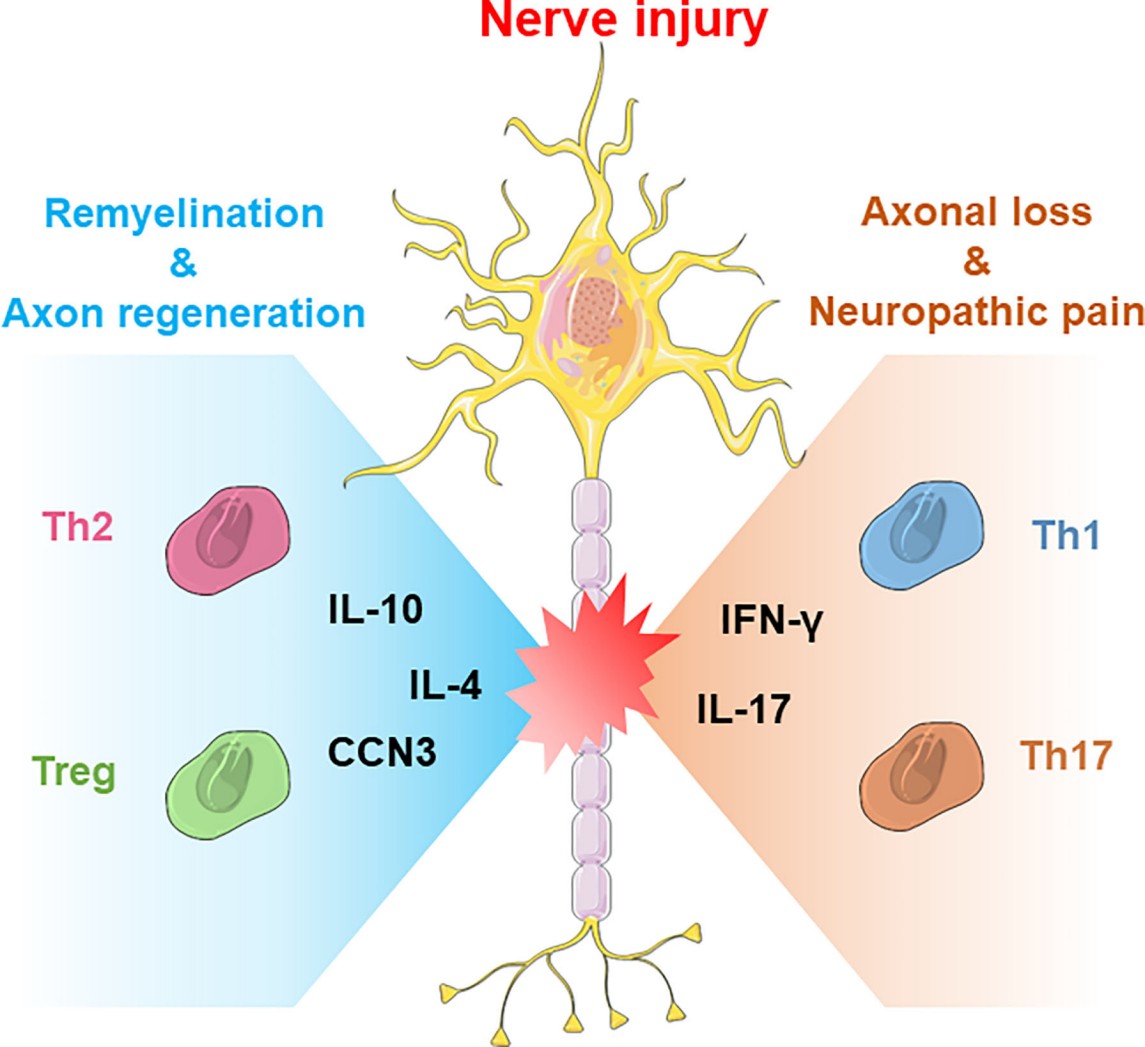
The neuroprotective and neurodestructive impacts of T-cell subsets in CNS/PNS nerve injury. Generally, Th2 cells and Treg cells produce anti-inflammatory cytokines such as IL-4 or IL-10 to promote nerve repair, whereas Th1 and Th17 cells produce IFN-γ or IL-17 to boost acute inflammatory responses.

In addition to immunosuppression, Treg cells also have other functions in CNS pathology, such as promoting remyelination and facilitating neuroprotection. In a stroke model, Saino’s study indicated that Treg cells enhanced the migration of neural progenitors and the neurogenesis process to improve functional recovery after stroke (53). Wang’s study showed that administration of Treg cells into the mouse brain increased the proliferation of neural stem cells through IL-10 after ischemic stroke (54). Dombrowski found that Treg cells also promoted the differentiation of OPCs to further facilitate remyelination in the central nervous system by producing cellular communication network Factor 3 (CCN3) (55). In addition, recent studies have shown that Treg cells also promote the polarization of macrophages in the dorsal root ganglion (DRG) to accelerate axon regeneration and inhibit neuropathic pain after traumatic peripheral injury (**Figure 1**) (56). In contrast, Schwartz’s group found that depletion of Treg cells enhances the spontaneous T-cell-dependent protective response to facilitate neuronal survival after CNS axonal injury, which indicates that future therapies with controllable immunosuppression capacity are necessary to achieve a balance between neuroprotection and avoiding autoimmune disease (57, 58).

However, despite substantial evidence supporting the beneficial effects of Treg cells for nerve repair, some studies have also reported their detrimental effects. For example, Baruch’s report indicated that immunosuppression of phagocytes by Treg cells impaired plaque clearance in an Alzheimer’s disease (AD) mouse model (59). Meanwhile, Kleinschnitz’s study showed that the depletion of Treg cells could reduce stroke lesion volume and improve neurological function (60). Nevertheless, the differences between the effects of Treg cells in nerve injury indicate the dynamic roles held by Treg cells in different stages of the repair process and the complexities of Treg cell interactions with other immune or nerve cells in the microenvironment, where a deeper understanding should be made in future studies (**Table 1**).

**Table 1:** Summary of therapies based on T cells modulation for nerve repair.

Disease	Species	Implicated T cell subset	Main cytokines	T cell-mediated therapy	Reference
Traumatic brain injury	rat/mouse/human	Th1/Th17/Treg	IL-17/IFN-γ	Transplantation of hNSCs	(50–52, 55, 71)
Spinal cord injury	mouse/rat	Th2/γδ T	IFN-γ/IL-4/IL-10	Anti-Vγ4, anti–IFN-γ, or anti–TNF-α antibodies/transplantation of Th2 with neurotrophin-3/transplantation of bone marrow-derived M2 macrophages	(79, 87, 88)
Peripheral nerve injury	mouse/rat	Th1/Th2/Th17	TNF-α/IFN-γ/IL-4	Local injection of plasmids/transplantation of hWJ-MSCs/local injection of Treg cells around ANAs	(33, 38, 56, 81, 93)

## T Cell-Mediated Therapies for Nerve Injuries

Thermal, mechanical, chemical, or ischemic damage to the CNS or PNS results in neuronal loss and neuronal dysfunction, usually accompanied by sensorimotor impairment, which alters the quality of life of patients (61–64). For peripheral nerve injury, autologous transplantation is still the gold standard at present; however, there are limitations such as limited donor sources and mismatching structure/size (65, 66). Therefore, developing bioengineered artificial nerves becomes inevitable. In the past few decades, based on the development of biomedical materials, the use of artificial nerve grafts to repair damaged peripheral nerves has been extensively studied and has achieved remarkable results in repairing short-distance peripheral nerve transection injuries (67, 68). However, the effective repair of long-distance peripheral injuries using artificial nerve grafts remains a great challenge (69–72). For CNS injury, the current strategies are even limited to achieving desirable nerve repair and functional recovery (73–75). In addition to therapies that directly promote rapid axon growth, recent studies have focused on creating a favorable microenvironment at injury sites by regulating the immune response (76–78).

As the effect of T cells on nerve regeneration has been gradually explored, strategies for T-cell modulation have started to draw the attention of researchers (79, 80). For instance, by local injection of plasmids into the epineurium of injured rat sciatic nerve for upregulating vascular endothelial growth factor (VEGF) and fibroblast growth factor 2 (FGF2), Ruslan’s team found that the levels of pro-inflammatory cytokines such as tumor necrosis factor alpha and interleukin 12 (IL-12) were significantly reduced in peripheral blood, while the levels of epidermal growth factor, interleukin 2 (IL-2), monocyte chemoattractant protein 1, regulated upon activation, normal T-cell expressed and presumably secreted factor (RANTES) were greatly increased after 7 days post-injection of plasmid to promote angiogenesis and sciatic nerve regeneration (81). The capacity of mesenchymal stem cells to successfully regulate the immune response has been demonstrated in medical fields such as tissue regeneration and autoimmune diseases (82–84). For nerve regeneration, Aline’s team found that the levels of Treg cells significantly increased in the blood, lymph nodes, and neural infiltrating cells after administration of human Wharton’s glia-derived mesenchymal stem cells (hWJ-MSCs) to the injury site of the sciatic nerve. Meanwhile, the anti-inflammatory cytokines IL-4 and IL-10 were significantly upregulated in the lymph nodes and nerves of HWJ-MSC-treated mice to improve functional recovery due to hWJ-MSC-induced Treg cell development to regulate the balance of pro- and anti-inflammatory responses in the injured sciatic nerve (85).

For central nerve injury, Laura’s team found that transplantation of human neural stem cells (hNSCs) into a mouse model of demyelination experimental autoimmune encephalomyelitis during the chronic phase achieved successful remyelination and neuroinflammation suppression by increasing central Treg cells, demonstrating that Treg cells can directly interact with stem and progenitor cells within tissues to promote central nerve regeneration (86). In Sun’s research, rapid recruitment of γδ T cells into the injury site within 24 hours along with high expression of the inflammatory cytokine IFN-γ was found to impair recovery after SCI, where treatment with anti-Vγ4, anti–IFN-γ, or anti–TNF-α antibodies effectively improved functional recovery (87). Ma’s study demonstrated that adoptive transfer of bone marrow-derived M2 macrophages after SCI significantly decreased spinal cord lesion volume and improved locomotor function recovery in rats. The M2 macrophages infiltrated into the injured sites and produced anti-inflammatory cytokines such as IL-10 and TGF-β to increase the Th2 cell fraction to support nerve repair (88). Moreover, to prevent the development of multiple sclerosis after CNS damage, Villoslada’s team used recombinant human NGF to treat CNS inflammation and demyelination in EAE. NGF treatment reduced the production of interferon-γ and increased the production of interleukin 10 in the CNS, achieving a shift toward Th2 responses to promote myelin repair (89, 90).

Clinically, biomedical nerve grafts are usually required for repairing traumatic nerve defects. Recent studies have found that the behavior of T cells plays an important role in nerve defect repair using acellular nerve grafts (ANAs). Deng’s study found that insufficient accumulation of T cells within long-scale ANAs resulted in reduced expression of both IFN-γ and IL-4, which may limit functional recovery and nerve regeneration across the ANAs (91, 92). To further improve the efficacy of nerve repair using peripheral nerve allografts, Bushman’s team locally injected Treg cells around peripheral nerve allografts by encapsulating them in a degradable hydrogel of polyethylene glycol norbornene. Treg cells were released from the hydrogel and infiltrated the graft within 14 days, suppressed the host immune response, and promoted nerve regeneration in rats (93). These studies indicate that localized delivery of neutralizing antibodies or Treg cells to modulate the immune microenvironment combined with nerve grafts can be an effective strategy for nerve defect repair.

## Future Perspectives

Desirable nerve repair and functional recovery of traumatic nerve injuries remain great challenges in the clinic, and the immune response is essential in the pathological process of neural regeneration. However, there is a certain need for a deeper understanding of cellular immunity in the nervous system and how T cells contribute to the immune response after injury, especially in exploring the roles of different T-cell subsets at various stages of the repair process. Significantly, several achievements have been made for effectively modulating T cells in the immune response using biomaterials, stem cells, antibodies, or immunosuppressive drugs. The combination of biomedical scaffolds with cell therapy or spatiotemporal delivery of immunomodulators/cytokines/chemokines can provide great opportunities, which can be facilitated by further research into immunology.

## Author Contributions

XT and JL drafted the main body of this manuscript. YY modified the manuscript. QL, TH, HZ, and XC helped with the manuscript editing and discussions. All authors contributed to the article and approved the submitted version.

## Funding

This work was financially supported by the National Science Foundation of China (Project No: 31830028) and Jiangsu Provincial Key Medical Center.

## Conflict of Interest

The authors declare that the research was conducted in the absence of any commercial or financial relationships that could be construed as a potential conflict of interest.

## Publisher’s Note

All claims expressed in this article are solely those of the authors and do not necessarily represent those of their affiliated organizations, or those of the publisher, the editors and the reviewers. Any product that may be evaluated in this article, or claim that may be made by its manufacturer, is not guaranteed or endorsed by the publisher.
